# Genetic Characterization of the Rayed Pearl Oyster *Pinctada radiata* in the Eastern Adriatic Sea (Central Mediterranean)

**DOI:** 10.3390/genes17040397

**Published:** 2026-03-30

**Authors:** Mirela Petrić, Rino Stanić, Tena Ćurko, Biljana Apostolska, Antonela Sovulj, Mate Šantić, Željka Trumbić

**Affiliations:** 1 Department of Marine Studies, University of Split, 21000 Split, Croatia; tc0122@more.unist.hr (T.Ć.); ztrumbic@unist.hr (Ž.T.); 2 Faculty of Science, University of Zagreb, 10000 Zagreb, Croatia; rinostanic@hotmail.com; 3 Faculty of Science, University of Split, 21000 Split, Croatia; radja@pmfst.hr (B.A.); antonela.sovulj@pmfst.hr (A.S.); msantic@pmfst.hr (M.Š.)

**Keywords:** bivalves, non-indigenous, genetic diversity, *COI*, ITS2, phylogeography

## Abstract

Background/Objectives: Non-indigenous species are increasingly reshaping Mediterranean marine ecosystems, particularly under ongoing climate warming. The rayed pearl oyster *Pinctada radiata*, a thermophilic species originating from the Indo-Pacific region, is one of the earliest and most successful invaders in the Mediterranean Sea and has recently established populations in the Adriatic Sea. Methods: This study integrates preliminary shell morphometric data with molecular genetic analyses based on mitochondrial cytochrome c oxidase subunit I (*COI*) and nuclear internal transcribed spacer 2 (ITS2) markers to confirm species identity and examine patterns of genetic variation in comparison with other Mediterranean Sea regions and the Persian Gulf. Results: Phylogenetic analyses based on *COI* confirmed *P. radiata* as a distinct and well-supported monophyletic lineage, whereas the nuclear ITS2 marker showed limited resolution and interspecific overlap. Mediterranean and Adriatic populations showed low *COI* haplotype and nucleotide diversity and weak genetic structuring, consistent with recent colonization and secondary expansion, whereas Persian Gulf populations were more genetically diverse. Conclusions: Future studies should employ larger sample sizes and broader geographic sampling across both the Mediterranean Sea and the full native range of *P. radiata*, combined with high-resolution genome-wide nuclear markers, to better resolve connectivity and invasion dynamics.

## 1. Introduction

The Mediterranean Sea is one of the most invaded marine ecosystems globally, characterized by a high number of established non-indigenous species (NIS) [1,2], primarily introduced through human activities such as shipping, aquaculture, and the opening of the Suez Canal, which have enabled continuous introductions of Indo-Pacific biota. Secondary dispersal of NIS within the Mediterranean basin, driven by natural expansion from neighboring areas and facilitated by rising seawater temperature [3,4,5], appears to contribute to the northward expansion of species already established in the Ionian Sea, thereby representing an important pathway for subsequent introduction into the Adriatic Sea [6]. The widespread presence of alien species along Mediterranean coasts threatens native communities through competition, habitat modification, and disease transmission, often resulting in significant ecological disruption and economic loss [7,8,9]. Conversely, many marine invaders can have a positive effect on ecological processes, for example, as ecosystem engineers by creating novel habitats and increasing spatial complexity [10,11,12], as well as on economic sectors such as fisheries and aquaculture [13].

The rayed pearl oyster *P. radiata* (Leach, 1814) is a native Indo-Pacific species that has successfully invaded the Mediterranean Sea following its introduction through the Suez Canal from the Red Sea. It is considered the first Lessepsian migrant in the Mediterranean Sea, first reported from the Egyptian coast in 1874 [14]. *P. radiata* exhibits a pelagic larval phase of approximately three weeks [15], which may contribute to its dispersal potential; however, its broad distribution in the Mediterranean basin is likely the result of multiple factors, including broad environmental tolerance, particularly temperature. Since its first record, the species has established numerous self-sustaining populations across the eastern [16,17,18,19,20], central [16,19,21,22,23,24], and western basin [25,26]. *P. radiata* is highly adaptable to subtropical conditions and tolerant of pollution, thriving even in enclosed or contaminated habitats [13,27]. Its rapid spread and ecological adaptability, due to inherent phenotypic plasticity, raise concerns regarding potential competition with native bivalves and alteration of local benthic communities [28,29]; however, no major ecological impacts have yet been confirmed [30]. Moreover, *P. radiata*’s potential economic relevance in the Mediterranean Sea has become increasingly evident in recent years. Theodorou et al. [31] investigated its market distribution within the Greek shellfish sector, emphasizing its emerging role as a substitute for traditionally exploited shellfish species whose stocks have declined due to heatwaves and overexploitation. Similarly, Calabrese et al. [32] demonstrated *P. radiata*’s significant potential for sustainable commercial exploitation, both in pearl production and as a food resource, owing to its high levels of functional omega-3 polyunsaturated fatty acids (EPA and DHA) and low accumulation of toxic compounds.

The success of a non-indigenous species’ dispersal is driven by its ecological and biological characteristics as well as prevailing environmental conditions [12]. In addition to these ecological and biological determinants, evolutionary and genetic processes also play a pivotal role in shaping invasive species establishment and spread [33]. The interaction between ecological adaptability and genetic variability underpins the capacity of NIS to persist and expand into novel environments; therefore, investigating genetic diversity and evolutionary dynamics is critical for understanding colonization potential, geographic invasion patterns, lag phases, and management interventions [33]. In addition, molecular markers can reveal bottlenecks, multiple source contributions, and the spatial and temporal dynamics of spread [33]. Despite *P. radiata*’s ecological importance and commercial potential, many aspects of its invasion biology still remain poorly understood, particularly regarding its genetic population structure in the Mediterranean Sea. Barbieri et al. [34] provided the first analysis of its genetic diversity and phylogeographic structure, revealing low overall genetic diversity, differentiation between native and eastern Mediterranean Sea populations, and recent or ongoing demographic expansion within the basin. Recently, Aguilo-Arce et al. [35] investigated the genetic structure of pearl oyster populations in the western Mediterranean Sea, highlighting higher haplotype diversity in native Indo-Pacific populations compared to non-native ones. Both studies emphasized the need for additional genetic analyses across a broader range to clarify the invasion dynamics and evolutionary history of this species.

In the Adriatic Sea, *P. radiata* was first recorded in 1996, when a specimen attached to an oil platform transported from Sicily was found in the Bay of Trieste, northern Adriatic Sea [36]. However, no subsequent records from this area have been documented [37]. In Croatian waters, the pearl oyster was first reported in 2006 in the northern Adriatic Sea [38], followed by records from Mljet Island in 2015 [23] and Pelješac Peninsula in 2016 [39] in the eastern central Adriatic Sea. Further occurrences in the south Adriatic Sea were documented in Albanian waters near Saranda in 2010 [6] and Vlora Bay in 2014 [40], and in Montenegro in 2016 [41]. Recent detection in Lim Bay, northern Adriatic Sea [42], suggests that *P. radiata* is expanding its distribution range. All these findings demonstrate that the pearl oyster has crossed from a status of occasional records into an established breeding population along the shoreline of the eastern Adriatic Sea, raising important ecological and management questions regarding its invasion dynamics, genetic structure and potential impacts on native benthic communities.

Thus, to improve understanding of *P. radiata*’s establishment in the Adriatic Sea and to contribute to the broader assessment of its ecological and economic role in the Mediterranean Sea, this present study provides molecular identification of Adriatic specimens using two genetic markers, mitochondrial cytochrome c oxidase subunit I (*COI*) and nuclear internal transcribed spacer 2 (ITS2), and examines patterns of genetic variation in comparison with other Mediterranean regions. This approach will provide essential information for future management, monitoring, and evaluation of *P. radiata*’s commercial potential or control strategies in an increasingly warming Mediterranean Sea.

## 2. Materials and Methods

### 2.1. Sampling and Morphological Measurements

**Figure 1:**
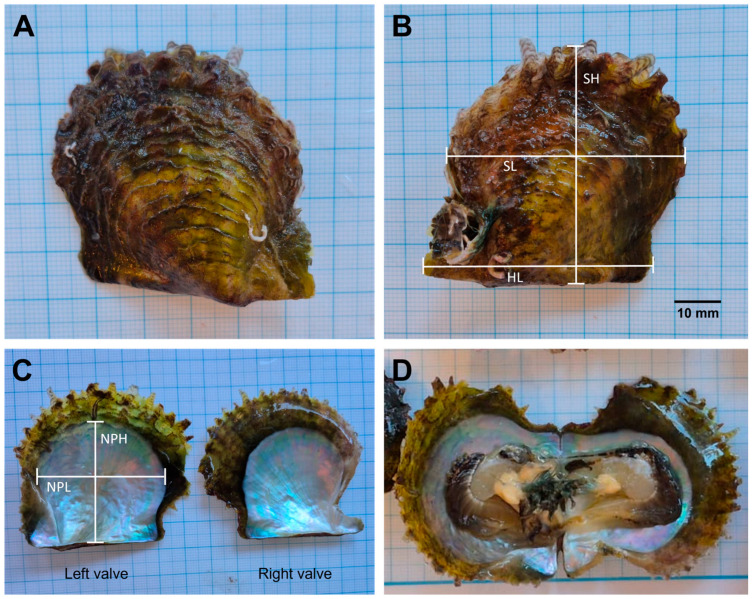
Rayed pearl oyster *P. radiata* collected from the eastern central Adriatic Sea: (**A**) external view—left valve; (**B**) external view—right valve; (**C**,**D**) internal view. Indicators on (**B**,**C**) illustrate the morphometric measurements: hinge length (HL), shell height (SH), shell length (SL), height of nacreous part (NPH) and length of the nacreous part (NPL).

**Figure 2:**
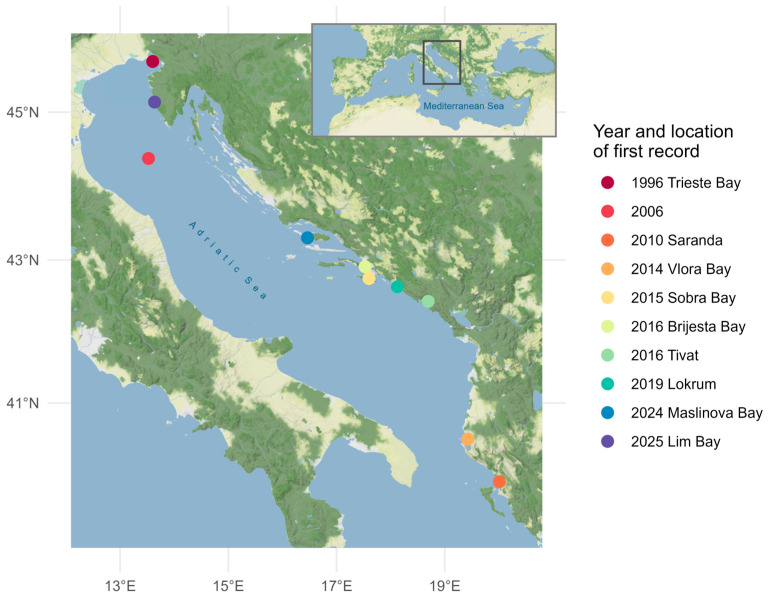
Sampling location for the rayed pearl oyster *P. radiata* in the eastern central Adriatic Sea. The sampling site in this study is at Maslinova Bay, Island Brač (43°18′13′′ N/16°27′47′′ E). Additional colored markers denote previously reported findings of *P. radiata* in the Adriatic Sea: 1996 Trieste Bay [36], 2006 [38], 2010 Saranda [6], 2014 Vlora Bay [40], 2015 Sobra Bay [23], 2016 Brijesta Bay [39], Tivat [41], 2019 Lokrum [24], 2024 Maslinova Bay (this study), 2025 Lim Bay [42]. Full details for all records are provided in Supplementary Table S1. The maps were produced using the ggmap package v4.0.0 [43] for R and tiles by © Stadia Maps (https://stadiamaps.com/, accessed on 27 January 2026), © Stamen Design (https://stamen.com/, accessed on 27 January 2026), © OpenMapTiles (https://openmaptiles.org/, accessed on 27 January 2026) and © OpenStreetMap (https://www.openstreetmap.org/#map=5/39.723/19.314, accessed on 27 January 2026). This figure is openly licensed via CC BY-NC-ND.

Nineteen individuals of the rayed pearl oyster *P. radiata* (Figure 1) were collected from Maslinova Bay (43°18′13′′ N/16°27′47′′ E), Island Brač (eastern central Adriatic Sea), at depths ranging from 5 to 12 m (Figure 2). Sampling was conducted in October 2024 (N = 10) and October 2025 (N = 9). Specimens were collected by professional SCUBA divers from the weights anchoring the fish cages at an aquaculture facility. Following collection, individuals were frozen and transported to the laboratory for further analyses.

Specimens were preliminarily identified as, *P. radiata,* based on shell morphology and external characteristics, following published species descriptions and records [39]. In the laboratory, individuals were thawed at room temperature, and epibionts were carefully removed from the shell surfaces. The total weight (TW) of each individual and the flesh weight without the byssus (FW) were measured using an analytical balance with a precision of 0.001 g. The shells were then opened, and a small portion of the adductor muscle was excised and preserved in 96% ethanol for subsequent molecular analyses. The remaining soft tissue was removed, and the shells were left to air-dry at room temperature. Once dried, morphometric parameters were measured following Tlig-Zouari et al. [21]. For each individual, shell width (SW), hinge length (HL), shell height (SH), shell length (SL), height of nacreous part (NPH), and length of the nacreous part (NPL) were measured using a digital caliper with a precision of ±0.01 mm. The condition index (CI) was calculated as the ratio of flesh weight to dry shell weight [44]. Age was not directly determined in this study. Instead, approximate age classes were inferred from shell height measurements by comparing the observed size distribution with previously published growth estimates and size–age relationships for *P. radiata* reported for Mediterranean Sea populations [18,22].

### 2.2. DNA Extraction, PCR Amplification and Sequencing

Genomic DNA was extracted from the adductor muscle following a modified protocol of Martínez et al. [45]. Tissue samples were digested overnight at 55 °C in cell lysis buffer (0.2% SDS, 0.01 M Tris-Base, 0.01 M EDTA, 0.15 M NaCl (Sigma-Aldrich, Steinheim Germany)) with proteinase K (0.2 mg/mL) (Sigma-Aldrich, Steinheim Germany). After digestion, 5 M NaCl was added to reach a final concentration of 2.2 M, and samples were cooled at 4 °C for 10 min. Proteins were removed by centrifugation (13,000 rpm, 10 min), and DNA was precipitated from the supernatant with an equal volume of isopropanol, followed by centrifugation (13,000 rpm, 10 min). The DNA pellet was washed with 75% ethanol, dried at 37 °C, and resuspended in 20–40 µL of TE buffer (0.01 M Tris-HCl, 0.0125 M EDTA, pH 8.0), depending on yield.

Partial sequences of the mitochondrial cytochrome c oxidase subunit I gene (*COI*, 710 bp) and the nuclear ribosomal internal transcribed spacer 2 with partial 5.8S and 28S ribosomal RNA genes (ITS2, 900 bp) were amplified using the primer pairs LCO1490: 5′-GGTCAACAAATCATAAAGATATTGG-3′ and HCO2198: 5′-TAAACTTCAGGGTGACCAAAAAATCA-3′ [46], and ITS2ModA: 5′-GCTTGCGGAGAATTAATGTGAA-3′ and ITS2ModB: 5′-GGTACCTTGTTCGCTATCGGA-3′ [47], respectively. PCR reactions (25 µL) contained 1× PCR buffer, 1.5 mM MgCl_2_, 0.2 mM dNTPs, 0.4 µM of each primer, 1 U GoTaq^®^ G2 Hot Start Polymerase (Promega Corporation, Madison, WI, USA), 50 ng of DNA template, and DNAse/RNAse-free water. For *COI*, PCR amplification was performed with an initial denaturation at 95 °C for 2 min; followed by 40 cycles of denaturation at 95 °C for 30 s, annealing at 45 °C for 90 s, and extension at 72 °C for 60 s; with a final extension at 75 °C for 5 min. For ITS2, PCR conditions consisted of an initial denaturation at 95 °C for 2 min; followed by 35 cycles of denaturation at 95 °C for 30 s, annealing at 50 °C for 30 s, and extension at 72 °C for 60 s; with a final extension at 75 °C for 5 min. Products were visualized on 1% agarose gels and sequenced with the ABI PRISM BigDye Terminator Kit (Applied Biosystems, Waltham, MA, USA) on an ABI 3100 sequencer in the forward direction. The service was outsourced to the Macrogen Europe sequencing laboratory (Amsterdam, The Netherlands) and Microsynth AG (Balgach, Switzerland).

### 2.3. Genetic Analysis

DNA sequences were processed, quality trimmed and aligned using the MUSCLE algorithm implemented in MEGA v12 [48]. The obtained *COI* and ITS2 sequences were deposited in GenBank (https://www.ncbi.nlm.nih.gov/genbank (accessed on 22 December 2025)) under accession numbers PX736884-PX736897 and PX740674-PX740688, respectively. Publicly available sequences of the genus *Pinctada* were retrieved from GenBank and included in the analyses of genetic diversity and phylogenetic relationships; these sequences represent populations from the Persian Gulf, Mediterranean Sea, Indo-Pacific, Pacific, and Atlantic regions. Detailed information on sequence origin and accession numbers is provided in Supplementary Tables S2 and S3.

Phylogenetic relationships were inferred using the maximum likelihood (ML) method implemented in IQ-TREE 2 [49], as installed on Padobran, a computational high-performance platform provided by the University of Zagreb Computing Centre SRCE. For the *COI* dataset, sequences were partitioned by codon position [50]. Model selection based on the Bayesian Information Criterion (BIC), as implemented in ModelFinder [51] in IQ-TREE 2, identified HKY+F+G4 as the best-fit substitution model for codon positions 1 and 2, and TN + F as the best-fit model for codon position 3. For the ITS2 dataset, model selection was performed without partitioning, and TPM3 + R2 was identified as the best-fit substitution model. Branch support was assessed using 1000 standard non-parametric bootstrap replicates. Phylogenetic trees were rooted using *Magallana gigas* (Thunberg, 1793) as the outgroup (*COI*: PQ739439; ITS2: FJ544290) and were visualized and graphically edited using FigTree v1.4.4 (http://tree.bio.ed.ac.uk/software/figtree, accessed on 8 February 2026). Pairwise genetic distances between *Pinctada* species were calculated using *p*-distance with MEGA v12 [48].

Genetic diversity was assessed using DnaSP v6.12.03 [52] by estimating the number of haplotypes (H), haplotype diversity (Hd), number of polymorphic sites (S), nucleotide diversity (π) [53], and the mean number of pairwise differences (k) [54]. Haplotype relationships were visualized using a median-joining haplotype network constructed in PopART v1.7 [55]. Pairwise genetic differentiation among populations was estimated using the fixation index *Φ_ST_*, as implemented in ARLEQUIN v3.5.2.2 2 [56], based on the Tamura–Nei distance model without gamma correction. The statistical significance of pairwise *Φ_ST_* values was evaluated using the exact test of population differentiation with 100,000 Markov chain steps, and significance levels were adjusted for multiple comparisons using the Bonferroni correction. Demographic history and deviations from neutral evolution were assessed using Tajima’s *D* [54] and Fu’s *Fs* [57] statistics. Demographic expansion was further evaluated using mismatch distribution analysis under a sudden expansion model, including estimation of the raggedness (r) index [58] and the expansion parameter tau (τ) with a 95% confidence interval. All analyses were conducted in ARLEQUIN v3.5.2.2 [56].

## 3. Results

All collected individuals keyed out morphologically as *P. radiata* according to the diagnostic characters provided in the relevant taxonomic descriptions. This morphological identification was consistent across specimens and was further confirmed by molecular analyses, which assigned all individuals to *P. radiata*.

Total shell weight (TW) of the analyzed *P. radiata* individuals ranged from 4.53 to 29.14 g (mean ± SD = 14.22 ± 6.70). Shell height (SH) varied between 31.43 and 68.09 mm (45.03 ± 8.96), while shell length (SL) ranged from 30.45 to 68.56 mm (45.31 ± 9.46). The condition index (CI) ranged from 43.56 to 73.21 (57.68 ± 8.79). Descriptive statistics for all shell morphometric parameters are presented in Table 1.

Based on published size–age relationships for *P. radiata*, the observed shell size distribution suggests that most analyzed individuals are approximately two to four years old. Although based on a small number of specimens (N = 19), the shell morphometric characteristics indicate the presence of a well-established and mature population in the eastern Adriatic Sea.

### 3.1. Phylogenetic Analysis

From the 19 individuals of *P. radiata* sampled in this study, 14 high-quality *COI* sequences and 15 high-quality ITS2 sequences were obtained and retained for further analysis. The initial *COI* alignment generated from our sequences was 560 bp in length, while the ITS2 alignment comprised 559 bp. For phylogenetic analyses including the global dataset, the *COI* sequences were trimmed to 397 bp and the ITS2 sequences to 559 bp to ensure overlap with the available reference sequences. The final global dataset used for phylogenetic reconstruction of the genus *Pinctada* therefore comprised alignments of 397 bp for *COI* and 764 bp for ITS2.

**Figure 3:**
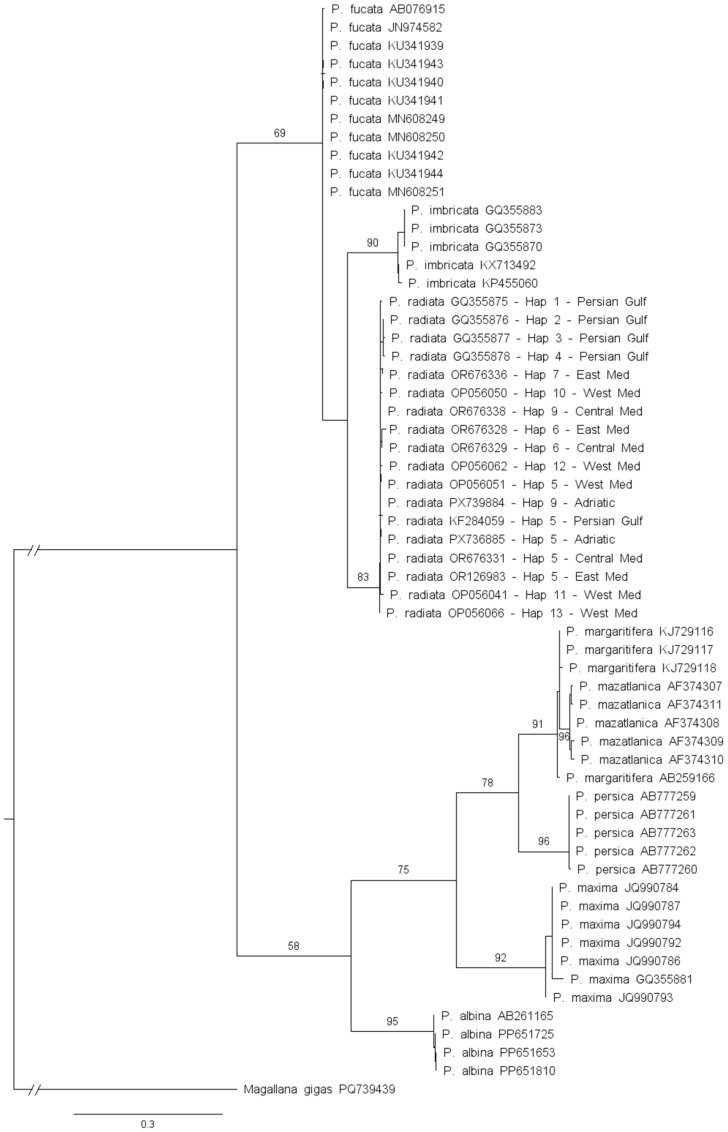
Maximum Likelihood phylogenetic analysis based on *COI* sequences of *Pinctada* species. Bootstrap support values (>50%) are shown at the nodes. Haplotypes of *P. radiata* from the Adriatic Sea, Mediterranean Sea regions (east, central and west), and the Persian Gulf are indicated on the terminal branches. *M. gigas* was used as an outgroup. The scale bar represents substitutions per site. Details of haplotypes and corresponding GenBank accession numbers are provided in Supplementary Table S4.

Maximum Likelihood phylogenetic analysis of *COI* sequences resolved *P. radiata* as a distinct monophyletic clade supported by a bootstrap value of 83% (Figure 3). Adriatic Sea specimens clustered within this clade together with sequences from the eastern, central, and western Mediterranean Sea and the Persian Gulf, confirming their taxonomic identity. Within the genus, distinct clusters were recovered for *Pinctada fucata* (A. A. Gould, 1850), *Pinctada imbricata* Röding, 1798, *Pinctada albina* (Lamarck, 1819), *Pinctada maxima* (Jameson, 1901), *Pinctada persica* (Jameson, 1901), *Pinctada margaritifera* (Linnaeus, 1758), and *Pinctada mazatlantica* (Hanley, 1856). Most interspecific relationships received moderate-to-high bootstrap support (>50%), with several nodes showing strong support (e.g., bootstrap values ≥ 90%). The reconstructed phylogenetic topology was also consistent with interspecific and intraspecific genetic distances observed for the *COI* gene (Table 2). Intraspecific mean uncorrected *p*-distances (range 0.05–1.42%) were substantially lower than interspecific distances (2.56–28.71%) and did not overlap.

**Figure 4:**
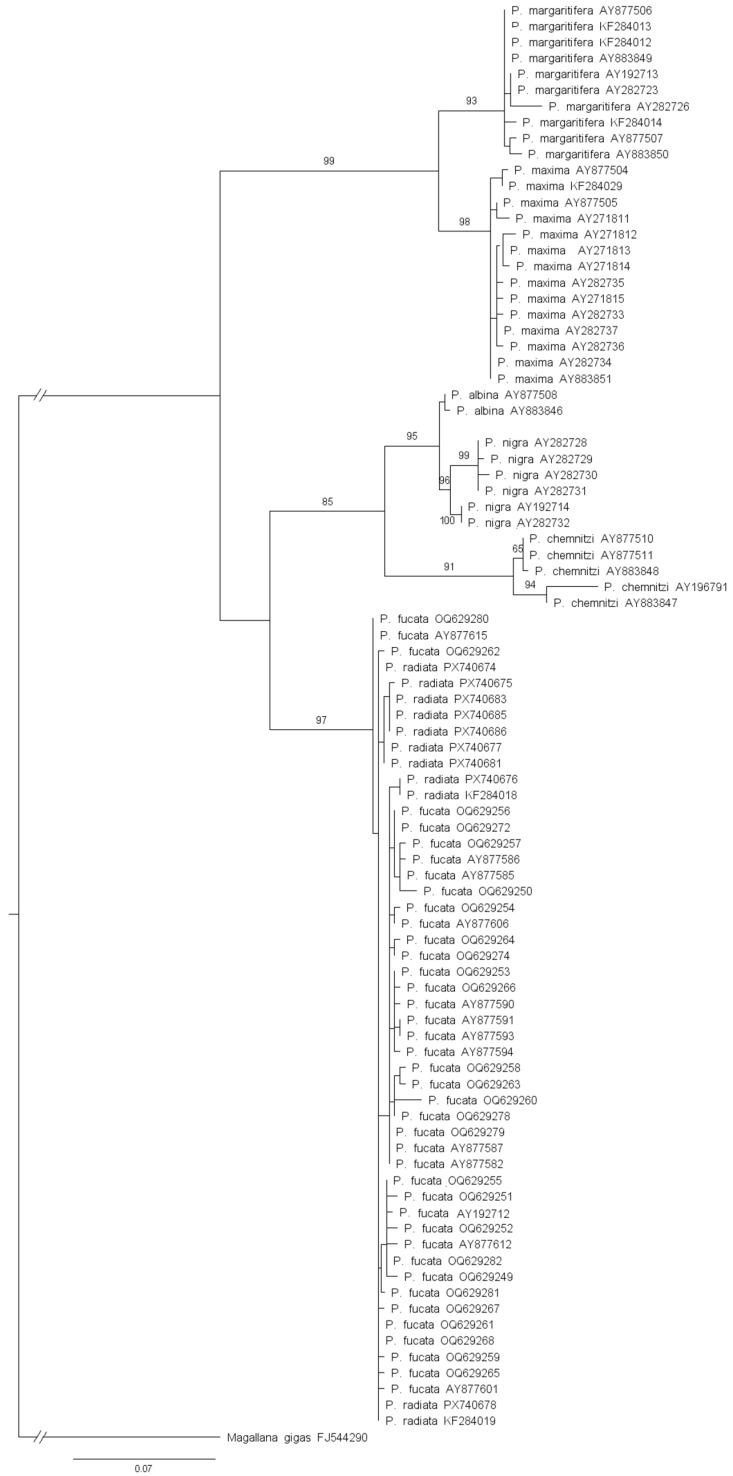
Maximum Likelihood phylogenetic analysis based on ITS2 sequences of *Pinctada* species. Bootstrap support values (>50%) are shown at the nodes. *M. gigas* was used as an outgroup. The scale bar represents substitutions per site. Adriatic *P. radiata* sequences are under GenBank accession numbers PX740674-PX740688; detailed information regarding their corresponding haplotypes is provided in Supplementary Table S5.

Maximum Likelihood phylogenetic analysis based on ITS2 sequences clustered *P. radiata* together within a single clade (Figure 4). Adriatic *P. radiata* grouped with other *P. radiata* sequences and did not form a separate lineage. Within the *P. radiata* clade, sequences were interspersed with those of *P. fucata*, and no clear sub-structuring or region-specific clustering was observed. Internal nodes within this clade generally showed low to moderate bootstrap support, indicating limited resolution of intra- and interspecific relationships based on the ITS2 marker for these two species. Other *Pinctada* species formed well-supported, species-specific clades, with high bootstrap support. Phylogenetic relationships were further supported by patterns of inter- and intra- specific genetic distances (Table 3). Interspecific mean uncorrected *p*-distances for the ITS2 gene ranged from 0.92 to 15.05%, which overlapped with the observed range of intraspecific distances, 0.22–2.15%. Namely, only the distance between *P. radiata* and *P. fucata* was much smaller than other interspecific distances, confirming poor resolution of ITS2 for this *Pinctada* species pair.

### 3.2. Genetic Analysis

Analysis of DNA polymorphism revealed contrasting patterns of genetic variation between the two markers, as expected. The *COI* dataset exhibited low levels of genetic variation, with only a single polymorphic site, resulting in two haplotypes. Consequently, haplotype diversity was low (Hd = 0.363 ± 0.13 SD), with a variance of 0.01695, and low nucleotide diversity (π = 0.00065). The mean number of pairwise differences was also correspondingly low (k = 0.363). In contrast, the ITS2 dataset showed higher genetic variability, with 13 polymorphic sites defining nine haplotypes. Haplotype diversity was high (Hd = 0.848 ± 0.09 SD), with a variance of 0.00772, while nucleotide diversity was moderate (π = 0.00676). The mean number of pairwise differences (k) was 3.771. As ITS2 demonstrated poor taxonomic resolution at the species level, only *COI* was used for the analyses of genetic diversity and differentiation of *P. radiata*.

DNA polymorphism analysis of *COI* sequences in the expanded dataset with sequences from the database of *P. radiata* from other geographic locations revealed heterogeneous genetic diversity (Table 4). The overall dataset (N = 59) contained 19 polymorphic sites and 13 haplotypes, resulting in moderate haplotype diversity (Hd = 0.511 ± 0.078) and low nucleotide diversity (π = 0.0026). The mean number of pairwise differences across all samples was 1.247, reflecting generally low mitochondrial sequence divergence.

Among Mediterranean Sea populations, genetic diversity was relatively low and heterogeneous. The Adriatic Sea (N = 14) and central Mediterranean Sea (N = 6) populations showed the lowest variation, with one polymorphic site and two haplotypes each, while the eastern Mediterranean Sea population (N = 9) displayed comparatively higher diversity, with three polymorphic sites and four haplotypes. Despite a larger sample size, the western Mediterranean Sea population (N = 22) showed relatively low haplotype and nucleotide diversity, indicating dominance of a limited number of haplotypes. In contrast to Mediterranean Sea populations, the Persian Gulf population (N = 8) showed higher haplotype diversity and nucleotide diversity, containing ten polymorphic sites and five haplotypes.

**Figure 5:**
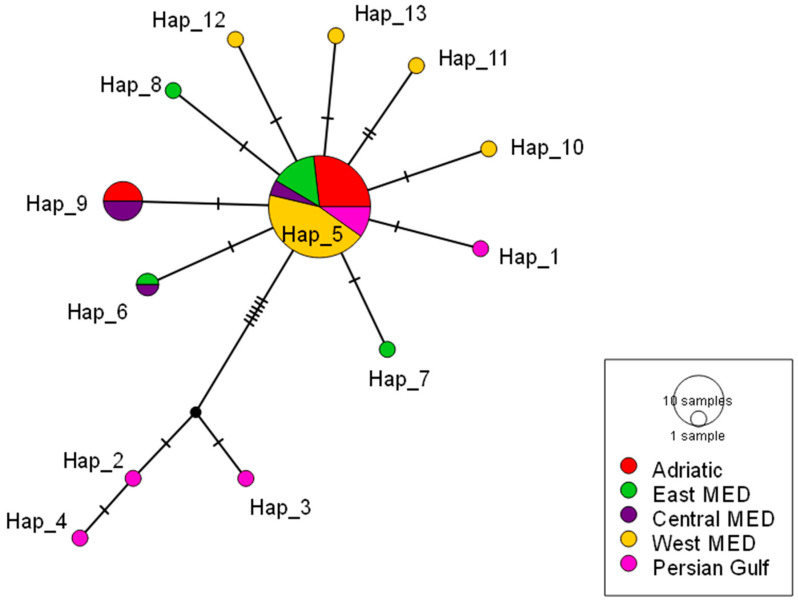
Median-joining haplotype network of *P. radiata COI* sequences from different geographic regions: Adriatic Sea (red), eastern Mediterranean Sea (green), central Mediterranean Sea (purple), western Mediterranean Sea (yellow) and Persian Gulf (pink). The size of each circle is proportional to the number of individuals sharing a given haplotype. Bars on branches indicate the number of mutational steps separating haplotypes, and black dots represent missing (unsampled) haplotypes.

These patterns of genetic diversity are consistent with the structure observed in the median-joining haplotype network (Figure 5), which is characterized by a dominant, centrally positioned haplotype (Hap 5) shared across all investigated regions and a star-like topology indicative of recent demographic expansion. Most Mediterranean Sea haplotypes differed from Hap 5 by one or a few mutational steps, while Persian Gulf haplotypes formed a distinct, peripheral cluster separated by multiple mutations. Additional region-specific haplotypes (Hap 6 and Hap 9) were associated with the Adriatic Sea, eastern and central Mediterranean Sea, and displayed limited sharing among populations.

Analysis of molecular variance (AMOVA) revealed a low level of overall genetic structuring across the analyzed populations of *P. radiata* (Table 5). The overall fixation index (*Φ_ST_*) of 0.245 showed that 24.5% of the total genetic variation occurred among populations and 75.5% within populations. The result of the exact test was statistically significant (*p* = 0.00377), supporting the inference of a non-random partitioning of genetic variation among geographic groups. However, pairwise comparisons did not reveal statistically significant differentiation between individual population pairs. The Persian Gulf population showed the highest *Φ_ST_* values in comparison with other regions, particularly with the western Mediterranean Sea (*Φ_ST_* = 0.393) and Adriatic Sea populations (*Φ_ST_* = 0.322). The lowest *Φ_ST_* values were observed among Mediterranean populations, including the eastern and western Mediterranean Sea populations (*Φ_ST_* = 0.022) and between Adriatic Sea and central Mediterranean Sea populations (*Φ_ST_* = 0.059).

The results of neutrality tests revealed heterogeneous demographic patterns among the examined *P. radiata* populations (Table 6). Tajima’s *D* values were positive, but not statistically significant for the Persian Gulf, Adriatic Sea and central Mediterranean Sea populations, indicating no departure from neutral expectations in these regions. However, the eastern and western Mediterranean Sea populations showed significant deviation from neutrality. Fu’s *Fs* statistics supported these inferences, with obtained non-significant positive or weak negative values for the Persian Gulf, Adriatic Sea, and central Mediterranean Sea populations, and significantly negative values for the eastern and western Mediterranean Sea populations.

For most populations, the observed DNA sequence mismatch distributions did not significantly deviate from expectations under a sudden demographic expansion model, as indicated by non-significant SSD and raggedness index values. The Persian Gulf population showed non-significant SSD and *r* values, and a higher τ value, suggesting an older demographic history. The populations within the Mediterranean basin also showed non-significant SSD and *r* values, consistent with demographic stability or recent expansion. The Adriatic Sea population exhibited a significant SSD value; however, there was a non-significant *r* index, indicating a deviation from the expansion model that may reflect recent demographic fluctuations.

## 4. Discussion

### 4.1. Morphometrics and Condition Index

Morphometric data were used primarily to characterize the size and maturity of the analyzed *P. radiata* specimens rather than to infer population structure. Observed shell height (31.43–68.09 mm; mean = 45.03) and shell length (30.45–68.56 mm; mean = 45.31) are in the range reported for established Mediterranean Sea populations [18,21,22,23,24,59,60]. Based on published growth estimates, *P. radiata* typically reaches shell heights of 40–50 mm within two to three years and exceeds 60 mm after three to five years under Mediterranean conditions, depending on environmental factors such as temperature and productivity [18,22]. Accordingly, the individuals analyzed here likely represent reproductively mature size classes, as *P. radiata* typically reaches sexual maturity at shell heights of approximately 17 mm [21], confirming the species as a stable, established component of the Adriatic Sea benthic fauna.

The condition index (CI), widely used as an indicator of physiological status in bivalves [61], ranged from 43.56 to 73.21 (mean = 57.68) in this study. These values are consistent with those reported for *P. radiata*, where CI varies in response to seasonal and environmental conditions [62,63]. Although comparisons among studies require careful normalization and consideration of methodological differences, comparable CI ranges have been reported for *P. radiata* and other bivalves, including the European flat oyster *Ostrea edulis* and the Mediterranean mussel *Mytilus galloprovinicialis*, where values reflect trophic conditions and reproductive cycles [64,65].

Given the limited sample size and restricted size range, no conclusions regarding population structure, growth patterns, or age distribution can be drawn. However, the observed morphometric characteristics and CI values indicate that the analyzed individuals are in good physiological condition.

As stated by Moutopoulos et al. [18], the limited availability of population-level abundance and age-and-growth data for *P. radiata* hampers effective stock assessment and the development of fisheries management strategies. Given the long-standing shellfish aquaculture tradition in Croatia and the confirmed establishment of *P. radiata* populations (e.g., Mljet Island), targeted studies on population dynamics, age structure, growth, abundance and spatial distribution across the Adriatic basin are strongly recommended. Such research would be essential to evaluate the species’ potential for controlled harvest or aquaculture production, as well as to assess its ecological role and possible interactions with native benthic communities.

### 4.2. Phylogenetics

The taxonomy and correct species delineation are somewhat complex for pearl oysters due to their high morphological similarity, pronounced phenotypic environmental plasticity and wide geographic range [19,66,67]. Notably, the three species *P. radiata*, *P. fucata*, and *P. imbricata* have been considered a species complex, or subspecies with shallow levels of divergence [23,34,66,68]. In addition to variable shell morphology characteristics and intraspecies variation, this was also linked to the choice of phylogenetic molecular marker used. Studies employing mitochondrial markers for phylogenetic inference, especially *COI* [19,20,35,67] have successfully resolved these three species as distinct taxonomic, genealogical, and evolutionary units, while less clear and ambiguous results were presented by those employing nuclear ribosomal markers for phylogeny or species identification [20,66,68,69]. This is also in accordance with our study where phylogenetic analysis using the *COI* gene, performed on 14 *P. radiata* individuals from the eastern central Adriatic Sea, confirmed *P. radiata* as a well-supported monophyletic lineage, clearly distinct from those of other congeners. In contrast, the nuclear ITS2 marker showed lower resolution and displayed interspersion of *P. radiata* and *P. fucata,* suggesting they might constitute a species complex. This pattern likely reflects the nature of each marker and evolutionary history of the species. ITS is a biparentally inherited, multi-copy gene array subject to concerted evolution and often evolves/changes more slowly between species than mitochondrial markers. As a nuclear marker with a larger effective population size, it may also sort more slowly among lineages. Mitochondrial DNA (e.g., *COI*) is typically maternally inherited, haploid, and non-recombining, and usually evolves more rapidly; however, in some bivalves, atypical inheritance systems and evidence of mitochondrial recombination have been reported [70]. Incongruences between mitochondrial- and nuclear-based phylogenies usually stem from incomplete lineage sorting, introgressive hybridization, or different modes of inheritance [66,67,71]. Given that Cuhna et al. [67] estimated the time of the most recent common ancestor of *P. radiata* and *P. imbricata* to 1.3 and 0.35 MYA, and *P. fucata* to 0.85 MYA, the most recent of all radiations within *Pinctada*, incomplete lineage sorting is the most probable explanation for the observed genetic patterns. Incomplete lineage sorting is an evolutionary process where ancestral polymorphisms persist, even when descendant lineages have diverged, causing gene trees to differ from the species tree. It is found in many taxa, especially if they diverged rapidly or have large effective population sizes and it can affect different parts of the genome differently [72]. This is also in accordance with our results, as ITS2 was more diverse overall, but could not reliably separate the *P. fucata*/*P. imbricata*/*P. radiata* complex, reflecting slow lineage sorting, whereas *COI*, despite comprising only two haplotypes in *P. radiata*, successfully reconstructed these species as monophyletic clades and resolved them as distinct species. It is hypothesized that the northward movement of the Australian plate throughout the Miocene [20.03–5.3 MYA] played an important role in shaping modern biogeographical patterns in the *Pinctada* genus. For instance, the uplift of the Indian archipelago might have isolated *P. radiata* of the Persian Gulf from species of the western Pacific Ocean (*P. fucata*) [67].

Considering the observed slow lineage sorting of ITS within *Pinctada*, and the fact that *COI*, despite limited variability, reflected species boundaries and shallow phylogeographic patterns, we used only *COI* to investigate the possible patterns of genetic differentiation of Adriatic Sea *P. radiata* in respect to other Mediterranean Sea specimens as well as individuals within its native range, the Persian Gulf.

Molecular identification of *P. radiata* from the Adriatic Sea was first reported by Nerlović et al. [39] (N = 1) and Gavrilović et al. [23] (N = 6); however, we were not able to find these sequences in public databases, thus limiting the opportunity for a broader comparative analysis with current samples within the Adriatic Sea. Results obtained in this study reveal low haplotype and nucleotide mitochondrial diversity for Adriatic Sea *P. radiata*, with only two haplotypes detected among 14 individuals, possibly due to the founder effect, typical of an invasive species. Similar or higher genetic diversity was detected for other Mediterranean Sea samples, with the highest diversity found in the eastern Mediterranean Sea. Higher haplotype and nucleotide diversity were recorded for the Persian Gulf, the native range of *P. radiata*. This general pattern of varying but generally lower genetic diversity in the Mediterranean Sea and higher genetic diversity in the Persian Gulf was also found in other studies [20,34,35]. In contrast, Gavrilović et al. [23] observed three *COI* haplotypes among six individuals in Sobra Bay in the Adriatic Sea, suggesting high haplotype diversity with low nucleotide diversity for *P. radiata*, more similar to what is observed in its native range. Sobra Bay (island of Mljet), situated in the central Adriatic Sea, is a well-documented locality for *P. radiata*, with its population repeatedly reported in the literature [23,24]. Beyond human-mediated transport via shipping and recreational boating, strong winter currents from the Strait of Otranto are also likely to facilitate the dispersal of biological material, as well as litter, into the Adriatic Sea and around the island of Mljet [23]. This seems to be an important gateway for the introduction of non-native species already established in the Mediterranean Sea [6]. It is possible that the dynamic hydrogeographic characteristics of the island of Mljet make it prone to multiple introductions of *P. radiata* from other areas with varying genetic characteristics, promoting the success of invasion and explaining higher genetic diversity [73]. However, further and more extensive studies are needed to confirm whether that is the case, or simply a question of sampling bias.

The loss of genetic diversity following an invasion is expected due to the founder effect, a form of genetic drift that arises when a new population is established by a few individuals, often leading to reduced diversity and atypical allele frequencies compared to the source population. Invasive populations are therefore expected to experience an increased probability of inbreeding and pronounced negative effects of genetic drift, possibly contributing to extinction risk. The fact that many show invasion success instead has been known as the ‘genetic paradox’ of invasive species [73,74]. Many invaders do not show a reduction in genetic diversity, in contrast to what is observed here for *P. radiata*. It is considered that large propagule pools combined with multiple introduction events contribute to the success of invasive species, as they reduce demographic stochastic effects and negative genetic consequences [73,74]. Alternatively, inbreeding might even have positive impacts on introduced populations by purging deleterious alleles, if there are any, resulting in fitness recovery [73]. The spread of *P. radiata* in the Mediterranean Sea is an ongoing process shaped by anthropogenic vectors, environmental factors with climate change playing an important role, and the biological, reproductive and adaptive capabilities of the species. The dominance of a single, widely shared haplotype across all regions, and a star-like haplotype network observed in our and other studies [34,35], is indicative of a recent population expansion. These patterns are frequently reported in marine bivalves with planktonic larvae and high dispersal potential, especially in species that have undergone recent range shifts or colonization events.

### 4.3. Management Implications

It is important to monitor biological invasions as they represent unique opportunities to track genetic changes within species facing new environments, observe footprints of adaptation and relate them to the new role that the species adopts in its non-native range. The choice of molecular markers is pivotal in this process. Although *COI* has proven informative and valuable in tracking species delineation in the genus *Pinctada*, its low genetic variability might make it less suitable for the study of population differentiation and sub-structuring within the Mediterranean Sea, if such occurs. Barbieri et al. [34] found a major genetic break between the Mediterranean Sea and Persian Gulf samples, and to a much lesser extent between Mediterranean Sea samples collected from the opposite western and eastern extremes of the basin, with no evidence of isolation by distance. In our study, overall *Φ_ST_* was significant between the western, central, and eastern Mediterranean Sea and the Persian Gulf; however, none of the pairwise *Φ_ST_* comparisons remained significant after correction for multiple testing. This result suggests the presence of weak but widespread population structure, where small genetic differences accumulate across populations but are insufficient to produce significant divergence in individual pairwise comparisons, or that the marker used has limited power to resolve these contrasts. As noted by Aguilo-Arce et al. [35], different studies have used varying portions of *COI* with relatively small overlap, meaning that combining all sequences in a single alignment would necessarily reduce the total alignment length to the overlapping regions. These authors have conducted multiple analyses based on different alignments and noticed that shorter sequences mean smaller numbers of haplotypes and smaller genetic distances. In our study, we opted to use only sequences that provided the longest and most informative alignment. However, this stresses the fact that some of the aspects of *P. radiata* diversity present in other sequences remain unexplored.

### 4.4. Future Research

In conclusion, although the sample size in our study was limited, to the best of our knowledge, the obtained sequences represent the first publicly available record for *P. radiata* from the Adriatic Sea. As such, they fill an important geographic gap and enable, for the first time, the inclusion of Adriatic Sea populations in Mediterranean-scale genetic comparisons. The placement of Adriatic Sea haplotypes within the broader Mediterranean Sea suggests connectivity with the wide Mediterranean Sea gene pool. Of taxonomic importance, our study also demonstrates the discrepancy between COI and ITS2 evolutionary dynamics, resulting in differing ability to resolve *P. radiata* as a distinct species. In this respect, COI can be used as a barcoding marker, while ITS2 shows probable signs of incomplete lineage sorting due to relatively shallow divergence. Future studies incorporating larger sample sizes, broader geographic coverage across both the Mediterranean Sea and the full Indo-Pacific range, and multiple high-resolution nuclear markers (e.g., microsatellites or genome-wide SNPs) will be essential to refine estimates of connectivity, resolve invasion pathways, and assess adaptive responses of *P. radiata* populations to environmental gradients.

## Figures and Tables

**Table 1:** Descriptive statistics of shell morphometric parameters (mm) of *P. radiata* (N = 19) from the eastern central Adriatic Sea.

Parameter	Mean	SD	Minimum	Maximum
Hinge length (HL)	44.76	6.880	30.69	55.75
Shell width (SW)	16.93	4.277	10.47	26.89
Shell height (SH)	45.03	8.961	31.43	68.09
Shell length (SL)	45.31	9.460	30.45	68.56
Nacreous part height (NPH)—left valve	35.58	6.904	23.84	51.30
Nacreous part length (NPL)—left valve	33.82	6.605	22.72	48.48
Nacreous part height (NPH)—right valve	32.46	6.512	22.15	47.12
Nacreous part length (NPL)—right valve	28.95	5.855	19.40	42.68

**Table 2:** Interspecies mean uncorrected *p*-distances (%) of the members of the *Pinctada* genus for the *COI* gene region. The number of base differences per site from averaging over all sequence pairs between groups is shown. Intraspecies distances (%) are placed on the diagonal and indicated in bold.

		1.	2.	3.	4.	5.	6.	7.	8.
**1.**	*P. albina*	**0.30**							
**2.**	*P. margaritifera*	22.82	**0.64**						
**3.**	*P. maxima*	22.30	17.75	**1.42**					
**4.**	*P. persica*	20.27	12.29	19.58	**0.10**				
**5.**	*P. fucata*	23.91	23.21	24.69	28.51	**0.05**			
**6.**	*P. imbricata*	24.38	28.65	24.43	27.38	12.56	**1.13**		
**7.**	*P. radiata*	24.32	26.12	25.41	26.93	9.18	13.81	**0.46**	
**8.**	*P. mazatlantica*	22.01	2.56	17.74	12.77	23.46	28.71	26.26	**0.92**

**Table 3:** Interspecies mean uncorrected *p*-distances (%) of the members of the *Pinctada* genus for the ITS2 gene region. The number of base differences per site from averaging over all sequence pairs between groups is shown. Intraspecies distances (%) are placed on the diagonal and indicated in bold.

		1.	2.	3.	4.	5.	6.	7.
**1.**	*P. radiata*	**0.59**						
**2.**	*P. fucata*	0.92	**0.92**					
**3.**	*P. albina*	10.45	10.46	**0.22**				
**4.**	*P. chemnitzi*	10.71	10.71	7.08	**1.92**			
**5.**	*P. margaritifera*	14.22	14.24	13.34	15.83	**0.77**		
**6.**	*P. maxima*	15.20	15.37	14.51	16.89	6.21	**0.78**	
**7.**	*P. nigra*	13.66	13.52	4.29	9.19	16.47	16.06	**2.15**

**Table 4:** Genetic diversity estimates for *COI* sequences of *P. radiata* populations from different geographic regions.

	N	S	H	Hd ± SD	π	k
**Adriatic Sea**	14	1	2	0.363 ± 0.130	0.00076	0.363
**Eastern Mediterranean Sea**	9	3	4	0.583 ± 0.183	0.00139	0.667
**Central Mediterranean Sea**	6	1	2	0.600 ± 0.129	0.00125	0.600
**Western Mediterranean Sea**	22	5	5	0.338 ± 0.128	0.00095	0.455
**Persian Gulf**	8	10	5	0.786 ± 0.151	0.00917	4.393
**Overall**	59	19	13	0.511 ± 0.078	0.00260	1.247

N—number of individuals, S—number of polymorphic sites, H—number of haplotypes, Hd—haplotype diversity, SD—standard deviation; π—nucleotide diversity, k—average number of nucleotide differences.

**Table 5:** Pairwise genetic differentiation (*Φ_ST_*) among geographic populations of *P. radiata* based on *COI* sequences.

	Persian Gulf	Adriatic	East Mediterranean	Central Mediterranean
**Adriatic Sea**	0.322			
**Eastern Mediterranean Sea**	0.248	0.090		
**Central Mediterranean Sea**	0.227	0.059	0.181	
**Western Mediterranean Sea**	0.393	0.079	0.022	0.287

**Table 6:** Results of neutrality test statistics and mismatch distribution analysis for the *COI* sequences of *P. radiata*.

	Neutrality Tests		Demographic Expansion Parameters		
	*D*	*Fs*	SSD	*r*	*τ* (SD_95%_)
**Persian Gulf**	0.6881	0.5153	0.0799	0.1429	6.9933
	*p* = 0.775	*p* = 0.573	*p* = 0.265	*p* = 0.768	(0.188–86.188)
**Adriatic Sea**	0.3244	0.64281	0.0045	0.2069	0.4897
	*p* = 0.769	*p* = 0.444	*p* = 0.000 ***	*p* = 0.639	(0–100.879)
**East** **Mediterranean Sea**	−1.5129	−1.8916	0.0297	0.1875	0.5148
	*p* = 0.051	*p* = 0.009 **	*p* = 0.087	*p* = 0.387	(0–60.667)
**Central** **Mediterranean Sea**	0.3106	−0.3041	0.0319	0.2222	1.1038
	*p* = 0.687	*p* = 0.234	*p* = 0.196	*p* = 0.476	(0–107.667)
**West** **Mediterranean Sea**	−1.9873	−3.1435	0.0001	0.2103	0.6421
	*p* = 0.005 **	*p* = 0.001 ***	*p* = 0.482	*p* = 0.849	(0–3.488)

*D*—Tajima’s statistic, *Fs*—Fu’s statistic, *r*—Harpending’s Raggedness index, SSD—sum of squared deviations, *τ*—tau, statistical significance *** *p* < 0.001, ** *p* < 0.01.

## Data Availability

The molecular data presented in this study are available in the National Center for Biotechnology Information archive under the GenBank Accession numbers PX736884-PX736897 and PX740674-PX740688.

## Supplementary Materials

The following supporting information can be downloaded at: https://www.mdpi.com/article/10.3390/genes17040397/s1, Table S1: Records of the rayed pearl oyster *P. radiata* in the Adriatic Sea; Table S2: List of GenBank Accession numbers of the genus *Pinctada COI* sequences used in the present study; Table S3: List of GenBank Accession numbers of genus *Pinctada* ITS2 sequences used in the present study; Table S4: *P. radiata COI* accession numbers from the present study and GenBank, with their haplotypes as presented in the phylogenetic tree in Figure 3; Table S5: Adriatic *P. radiata* ITS2 accession numbers from the present study, with their haplotypes as presented in the phylogenetic tree in Figure 4. References [75,76,77,78,79,80,81,82,83,84,85,86,87,88,89] are cited in the Supplementary Materials.
