# Supplementary material for: Genetic Characterization of the Rayed Pearl Oyster Pinctada radiata in the Eastern Adriatic Sea (Central Mediterranean)
Source: Genes (Basel). 2026 Mar 30;17(4):397. doi: 10.3390/genes17040397 (PMC13116804; doi:10.3390/genes17040397)
Supplement: Supplementary file 1 [file genes-17-00397-s001.zip › genes-4228116-supplementary.pdf]

Supplementary Materials

Table S1. Records of the rayed pearl oyster *Pinctada radiata* in the Adriatic Sea. (SL – shell length; SH – shell hight).

Author	Location	Year of finding	Number of individuals	Morphological assessment	Molecular assessment	Additional data
Vio and De Min, 1996	North Adriatic, Trieste Bay, Italy	1996	/	/	/	On an oil platform from the Sicily Channel in reparation in Trieste.
Dogan and Nerlović, 2008	North Adriatic, Croatia	2006	2	SL = 2.8 and 2.5 mm	/	Two juvenile specimens.
Katsanevakis et al., 2011	South Adriatic, Saranda, Albania	2010	1	/	/	One live specimen.
Gerovasileiou et al., 2017	South Adriatic, Vlora Bay, Albania	2014	/	/	/	Commonly observed alive at low depth in *Posidonia oceanica* meadows.
Nerlović et al., 2016	East Adriatic, Brijesta Bay, Croatia	2016	1	SL = 59.34 mm; SH = 61.37mm; HL = 56.43; SW = 19.67 mm	*COI*, 16sRNA sequences not publicly available	At 11 m depth, near the oyster farm.
Gavrilović et al., 2017	East Adriatic, island Mljet, Croatia	2015; 2017	6; 30	SH = 67.45 – 81.14; 30 ind. SH = 38.25 – 76.9	*COI*, sequences not publicly available	At 5 – 15 m depth, from the fish farm.
Petović and Mačić, 2017	South Adriatic, Tivat, Montenegro	2016	15	SH = 32 – 52 mm; average SH = 38.3 mm	/	At 5 m depth, on a newly constructed cement part of the port pier.
Bratoš Cetinić et al., 2023	East Adriatic, island Mljet, island Lokrum, Croatia	2019; 2021; 2022	48;	SH = 50.55 – 72.89 mm; SL = 46.66 – 71.15 mm	/	At 4 – 6 m depth, from the ropes of a fish farm
CIM IRB press	North Adriatic, Lim Bay, Croatia	2025	7	SL = 48 – 80 mm	/	/
Present study	East Adriatic, island Brač, Croatia	2024; 2025	10; 9	SH = 31.43 – 68.09; SL = 30.45 – 68.56 mm	*COI*, ITS2	At depths from 5 to 12 m, from an aquaculture facility.

Table S2. List of GenBank Accession numbers of genus *Pinctada COI* sequences used in the present study.

Species	Acc. No.	Locality	Region	Reference
Pinctada radiata	PX736884-PX736897	Maslinova Bay, island Brač	East Central Adriatic Sea	Present study
Pinctada radiata	GQ355875	United Arab Emirates	Persian (Arabian) Gulf	Cunha et al., 2011
Pinctada radiata	GQ355876	United Arab Emirates	Persian (Arabian) Gulf	Cunha et al., 2011
Pinctada radiata	GQ355877	United Arab Emirates	Persian (Arabian) Gulf	Cunha et al., 2011
Pinctada radiata	GQ355878	United Arab Emirates	Persian (Arabian) Gulf	Cunha et al., 2011
Pinctada radiata	KF284059	Ras Al Khaimah	Persian (Arabian) Gulf	Meyer et al., 2013
Pinctada radiata	KF284060	Ras Al Khaimah	Persian (Arabian) Gulf	Meyer et al., 2013
Pinctada radiata	KF284061	Ras Al Khaimah	Persian (Arabian) Gulf	Meyer et al., 2013
Pinctada radiata	KF284062	Ras Al Khaimah	Persian (Arabian) Gulf	Meyer et al., 2013
Pinctada radiata	OR126983	Kefalonia Island, Greece	Eastern Mediterranean	Papadopoulos et al., 2024
Pinctada radiata	OR126982	Evoikos Gulf, Greece	Eastern Mediterranean	Papadopoulos et al., 2024
Pinctada radiata	OR126981	Maliakos Gulf, Greece	Eastern Mediterranean	Papadopoulos et al., 2024
Pinctada radiata	OR676328	Cyprus, Polis	Eastern Mediterranean	Garzia et al., 2024
Pinctada radiata	OR676329	Italy,: Messina	Central Mediterranean	Garzia et al., 2024
Pinctada radiata	OR676330	Cyprus, Polis	Eastern Mediterranean	Garzia et al., 2024
Pinctada radiata	OR676331	Malta, Blue Grotto	Central Mediterranean	Garzia et al., 2024
Pinctada radiata	OR676332	Malta, Blue Grotto	Central Mediterranean	Garzia et al., 2024
Pinctada radiata	OR676333	Greece, Astypalea Island	Eastern Mediterranean	Garzia et al., 2024
Pinctada radiata	OR676334	Italy, Sassari, Olbia, Punta delle Saline	Western Mediterranean	Garzia et al., 2024
Pinctada radiata	OR676335	Greece, Rhodes, Lindos	Eastern Mediterranean	Garzia et al., 2024
Pinctada radiata	OR676336	Greece, Astypalea Island	Eastern Mediterranean	Garzia et al., 2024
Pinctada radiata	OR676337	Greece, Rhodes, Lindos	Eastern Mediterranean	Garzia et al., 2024
Pinctada radiata	OR676338	Italy, Messina	Central Mediterranean	Garzia et al., 2024
Pinctada radiata	OR676339	Italy, Briatico	Western Mediterranean	Garzia et al., 2024
Pinctada radiata	OR676340	Italy, Briatico	Western Mediterranean	Garzia et al., 2024

Pinctada radiata	OR676341	Italy, Taranto, Mar Piccolo di Taranto	Central Mediterranean	Garzia et al., 2024
Pinctada radiata	OR676342	Italy, Taranto, Mar Piccolo di Taranto	Central Mediterranean	Garzia et al., 2024
Pinctada radiata	OP056055	Cala Nova	Western Mediterranean	Aguilo-Arce et al., 2023
Pinctada radiata	OP056056	Can Pastilla	Western Mediterranean	Aguilo-Arce et al., 2023
Pinctada radiata	OP056059	Cala Gamba	Western Mediterranean	Aguilo-Arce et al., 2023
Pinctada radiata	OP056053	Cala Gamba	Western Mediterranean	Aguilo-Arce et al., 2023
Pinctada radiata	OP056051	Portixol	Western Mediterranean	Aguilo-Arce et al., 2023
Pinctada radiata	OP056046	Portixol	Western Mediterranean	Aguilo-Arce et al., 2023
Pinctada radiata	OP056045	Portixol	Western Mediterranean	Aguilo-Arce et al., 2023
Pinctada radiata	OP056039	Portixol	Western Mediterranean	Aguilo-Arce et al., 2023
Pinctada radiata	OP056050	Cala Nova	Western Mediterranean	Aguilo-Arce et al., 2023
Pinctada radiata	OP056038	Cala Nova	Western Mediterranean	Aguilo-Arce et al., 2023
Pinctada radiata	OP056054	S’Arenal	Western Mediterranean	Aguilo-Arce et al., 2023
Pinctada radiata	OP056044	S’Arenal	Western Mediterranean	Aguilo-Arce et al., 2023
Pinctada radiata	OP056049	S’Arenal	Western Mediterranean	Aguilo-Arce et al., 2023
Pinctada radiata	OP056052	S’Arenal	Western Mediterranean	Aguilo-Arce et al., 2023
Pinctada radiata	OP056042	S’Arenal	Western Mediterranean	Aguilo-Arce et al., 2023
Pinctada radiata	OP056041	S’Arenal	Western Mediterranean	Aguilo-Arce et al., 2023
Pinctada radiata	OP056047	S’Arenal	Western Mediterranean	Aguilo-Arce et al., 2023
Pinctada radiata	OP056067	S’Arenal	Western Mediterranean	Aguilo-Arce et al., 2023
Pinctada radiata	OP056048	S’Arenal	Western Mediterranean	Aguilo-Arce et al., 2023
Pinctada radiata	OP056058	S’Arenal	Western Mediterranean	Aguilo-Arce et al., 2023
Pinctada radiata	OP056043	S’Arenal	Western Mediterranean	Aguilo-Arce et al., 2023
Pinctada radiata	OP056040	Portixol	Western Mediterranean	Aguilo-Arce et al., 2023
Pinctada radiata	OP056057	Portixol	Western Mediterranean	Aguilo-Arce et al., 2023
Pinctada radiata	OP056062	Portixol	Western Mediterranean	Aguilo-Arce et al., 2023
Pinctada radiata	OP056064	Portixol	Western Mediterranean	Aguilo-Arce et al., 2023
Pinctada radiata	OP056065	Portixol	Western Mediterranean	Aguilo-Arce et al., 2023
Pinctada radiata	OP056066	Portixol	Western Mediterranean	Aguilo-Arce et al., 2023
Pinctada radiata	OP056063	Caló d’en Pellice	Western Mediterranean	Aguilo-Arce et al., 2023

Pinctada radiata	OP056068	Caló d’en Pellice	Western Mediterranean	Aguilo-Arce et al., 2023
Pinctada radiata	OP056061	Cala St. Antoni (Menorca)	Western Mediterranean	Aguilo-Arce et al., 2023
Pinctada radiata	OP056060	Riu pla (Menorca)	Western Mediterranean	Aguilo-Arce et al., 2023
Pinctada fucata	AB076915	NA	NA	Matsumoto, 2003
Pinctada fucata	JN974582	China	Pacific	Feng et al., 2015
Pinctada fucata	KU341939	China	Pacific	Feng et al., unpublished
Pinctada fucata	KU341940	China	Pacific	Feng et al., unpublished
Pinctada fucata	KU341941	China	Pacific	Feng et al., unpublished
Pinctada fucata	KU341942	China	Pacific	Feng et al., unpublished
Pinctada fucata	KU341943	China	Pacific	Feng et al., unpublished
Pinctada fucata	KU341944	China	Pacific	Feng et al., unpublished
Pinctada fucata	MN608249	China	Pacific	Lin, unpublished
Pinctada fucata	MN608250	China	Pacific	Lin, unpublished
Pinctada fucata	MN608251	China	Pacific	Lin, unpublished
Pinctada imbricata	GQ355883	Guadeloupe, NW Atlantic	Atlantic	Cunha et al., 2011
Pinctada imbricata	GQ355873	Guadeloupe, NW Atlantic	Atlantic	Cunha et al., 2011
Pinctada imbricata	GQ355870	Guadeloupe, NW Atlantic	Atlantic	Cunha et al., 2011
Pinctada imbricata	KX713492	Florida Keys	Atlantic	Combosch et al., 2016
Pinctada imbricata	KP455060	Caribbean Sea, W Atlantic	Atlantic	Pagenkopp et al., 2015
Pinctada albina	AB261165	Japan, Kagoshima, Amami	Pacific	Takakura, unpublished
Pinctada albina	PP651725	Pacific Ocean, South China Sea	Pacific	McIlroy et al., 2024
Pinctada albina	PP651653	Pacific Ocean, South China Sea	Pacific	McIlroy et al., 2024
Pinctada albina	PP651810	Pacific Ocean, South China Sea	Pacific	McIlroy et al., 2024
Pinctada margaritifera	AB259166	Japan, Okinawa	Pacific	Takakura, unpublish
Pinctada margaritifera	KJ729116	South Korea, Beomseom Island	Pacific	Oh et al., 2017
Pinctada margaritifera	KJ729117	Micronesia, Chuuk lagoon	Pacific	Oh et al., 2017
Pinctada margaritifera	KJ729118	France, Tahiti	Pacific	Oh et al., 2017
Pinctada persica	AB777259	Iran, Persian Gulf	Persian Gulf	Sharif Ranjbar et al., 2016
Pinctada persica	AB777260	Iran, Persian Gulf	Persian Gulf	Sharif Ranjbar et al., 2016
Pinctada persica	AB777261	Iran, Persian Gulf	Persian Gulf	Sharif Ranjbar et al., 2016

Pinctada persica	AB777262	Iran, Persian Gulf	Persian Gulf	Sharif Ranjbar et al., 2016
Pinctada persica	AB777263	Iran, Persian Gulf	Persian Gulf	Sharif Ranjbar et al., 2016
Pinctada maxima	GQ355881	Australia	Pacific	Cunha et al., 2011
Pinctada maxima	JQ990787	Indo-Australian Archipelago	Indo-Pacific	Lind et al., 2012
Pinctada maxima	JQ990794	Indo-Australian Archipelago	Indo-Pacific	Lind et al., 2012
Pinctada maxima	JQ990784	Indo-Australian Archipelago	Indo-Pacific	Lind et al., 2012
Pinctada maxima	JQ990793	Indo-Australian Archipelago	Indo-Pacific	Lind et al., 2012
Pinctada maxima	JQ990786	Indo-Australian Archipelago	Indo-Pacific	Lind et al., 2012
Pinctada maxima	JQ990792	Indo-Australian Archipelago	Indo-Pacific	Lind et al., 2012
Pinctada mazatlantika	AF374307	American Pacific coasts	Pacific	Arnaud et al., 2001
Pinctada mazatlantika	AF374311	American Pacific coasts	Pacific	Arnaud et al., 2001
Pinctada mazatlantika	AF374308	American Pacific coasts	Pacific	Arnaud et al., 2001
Pinctada mazatlantika	AF374309	American Pacific coasts	Pacific	Arnaud et al., 2001
Pinctada mazatlantika	AF374310	American Pacific coasts	Pacific	Arnaud et al., 2001
Magallana gigas	PQ739439	Netherlands	North Sea	Christodoulou et al., 2025

Table S3. List of GenBank Accession numbers of genus *Pinctada* ITS2 sequences used in the present study.

Species	Acc. No.	Locality	Region	Reference
Pinctada radiata	PX740674-PX740688	Maslinova Bay, island Brač	East Central Adriatic Sea	Present study
Pinctada radiata	KF284018	Ras Al Khaimah	Persian Gulf	Meyer et al., 2013
Pinctada radiata	KF284019	Ras Al Khaimah	Persian Gulf	Meyer et al., 2013
Pinctada fucata	AY877585	China: Daya Bay	Pacific	Yu and Chu, 2006
Pinctada fucata	AY877586	China: Daya Bay	Pacific	Yu and Chu, 2006
Pinctada fucata	AY877582	China: Sanya Bay	Pacific	Yu et al., 2006
Pinctada fucata	AY877587	China: Sanya Bay	Pacific	Yu et al., 2006
Pinctada fucata	AY877590	China: Beibu Bay	Pacific	Yu et al., 2006
Pinctada fucata	AY877591	China: Sanya Bay	Pacific	Yu and Chu, 2006
Pinctada fucata	AY877593	China: Sanya Bay	Pacific	Yu et al., 2006
Pinctada fucata	AY877594	China: Beibu Bay	Pacific	Yu et al., 2006
Pinctada fucata	AY877601	China: Beibu Bay	Pacific	Yu et al., 2006
Pinctada fucata	AY877606	Australia	Pacific	Yu et al., 2006
Pinctada fucata	AY877612	Japan	Pacific	Yu et al., 2006
Pinctada fucata	AY877615	Japan	Pacific	Yu et al., 2006
Pinctada fucata	OQ629249	China	Pacific	Shan et al., 2023
Pinctada fucata	OQ629250	China	Pacific	Shan et al., 2023
Pinctada fucata	OQ629251	China	Pacific	Shan et al., 2023
Pinctada fucata	OQ629252	China	Pacific	Shan et al., 2023
Pinctada fucata	OQ629253	China	Pacific	Shan et al., 2023
Pinctada fucata	OQ629254	China	Pacific	Shan et al., 2023
Pinctada fucata	OQ629255	China	Pacific	Shan et al., 2023
Pinctada fucata	OQ629256	China	Pacific	Shan et al., 2023
Pinctada fucata	OQ629257	China	Pacific	Shan et al., 2023
Pinctada fucata	OQ629258	China	Pacific	Shan et al., 2023

Pinctada fucata	OQ629259	China	Pacific	Shan et al., 2023
Pinctada fucata	OQ629260	China	Pacific	Shan et al., 2023
Pinctada fucata	OQ629261	China	Pacific	Shan et al., 2023
Pinctada fucata	OQ629262	China	Pacific	Shan et al., 2023
Pinctada fucata	OQ629263	China	Pacific	Shan et al., 2023
Pinctada fucata	OQ629264	China	Pacific	Shan et al., 2023
Pinctada fucata	OQ629265	China	Pacific	Shan et al., 2023
Pinctada fucata	OQ629266	China	Pacific	Shan et al., 2023
Pinctada fucata	OQ629267	China	Pacific	Shan et al., 2023
Pinctada fucata	OQ629268	China	Pacific	Shan et al., 2023
Pinctada fucata	OQ629272	China	Pacific	Shan et al., 2023
Pinctada fucata	OQ629274	China	Pacific	Shan et al., 2023
Pinctada fucata	OQ629278	China	Pacific	Shan et al., 2023
Pinctada fucata	OQ629279	China	Pacific	Shan et al., 2023
Pinctada fucata	OQ629280	China	Pacific	Shan et al., 2023
Pinctada fucata	OQ629281	China	Pacific	Shan et al., 2023
Pinctada fucata	OQ629282	China	Pacific	Shan et al., 2023
Pinctada martensii	AY192712	China	Pacific	He and Huang, 2023
Pinctada albina	AY877508	Australia	Pacific	Yu and Chu, 2006
Pinctada albina	AY883846	Australia	Pacific	Yu and Chu, 2006
Pinctada chemitzi	AY877511	China	Pacific	Yu and Chu, 2006
Pinctada chemitzi	AY877510	China	Pacific	Yu and Chu, 2006
Pinctada chemitzi	AY883847	China: Hong Kong	Pacific	Yu and Chu, 2006
Pinctada chemitzi	AY883848	China: Daya Bay, Guangdong	Pacific	Yu and Chu, 2006
Pinctada chemitzi	AY196791	China	Pacific	He et al., 2005
Pinctada maxima	AY883851	China: Sanya Bay, Hainan	Pacific	Yu and Chu, unpublished
Pinctada maxima	AY877504	China: Sanya Bay, Hainan	Pacific	Yu and Chu, 2006
Pinctada maxima	AY877505	China: Sanya Bay, Hainan	Pacific	Yu and Chu, 2006
Pinctada maxima	AY271811	China	Pacific	He et al., 2005

Pinctada maxima	AY27182	China	Pacific	He et al., 2005
Pinctada maxima	AY271813	China	Pacific	He et al., 2005
Pinctada maxima	AY271814	China	Pacific	He et al., 2005
Pinctada maxima	AY271815	China	Pacific	He et al., 2005
Pinctada maxima	AY282733	China	Pacific	He et al., 2005
Pinctada maxima	AY282734	China	Pacific	He et al., 2005
Pinctada maxima	AY282735	China	Pacific	He et al., 2005
Pinctada maxima	AY282736	China	Pacific	He et al., 2005
Pinctada maxima	AY282737	China	Pacific	He et al., 2005
Pinctada maxima	KF284029	Australia	Pacific	Meyer et al., 2013
Pinctada nigra	AY282728	China	Pacific	He et al., 2005
Pinctada nigra	AY282729	China	Pacific	He et al., 2005
Pinctada nigra	AY282730	China	Pacific	He et al., 2005
Pinctada nigra	AY282731	China	Pacific	He et al., 2005
Pinctada nigra	AY282732	China	Pacific	He et al., 2005
Pinctada nigra	AY192714	China	Pacific	He and Huang, 2023
Pinctada margaritifera	AY883849	China: Sanya Bay, Hainan	Pacific	Yu and Chu, unpublished
Pinctada margaritifera	AY883850	China: Sanya Bay, Hainan	Pacific	Yu and Chu, unpublished
Pinctada margaritifera	AY877506	China: Sanya Bay, Hainan	Pacific	Yu and Chu, 2006
Pinctada margaritifera	AY877507	China: Sanya Bay, Hainan	Pacific	Yu and Chu, 2006
Pinctada margaritifera	AY192713	China	Pacific	He and Huang, 2023
Pinctada margaritifera	AY282723	China	Pacific	He et al., 2005
Pinctada margaritifera	AY282726	China	Pacific	He et al., 2005
Pinctada margaritifera	KF284012	French Polynesia	Pacific	Meyer et al., 2013
Pinctada margaritifera	KF284013	French Polynesia	Pacific	Meyer et al., 2013
Pinctada margaritifera	KF284014	French Polynesia	Pacific	Meyer et al., 2013
Magallana gigas	FJ544290	Atlantic	Atlantic	Lazoski et al., 2011

Table S4. The *Pinctada radiata COI* accession numbers from the present study and GenBank, with their haplotypes as presented in the phylogenetic tree in Figure 3.

	COI Accession Number	N	Region
**Hap 1**	GQ355875	1	Persian Gulf
**Hap 2**	GQ355876	1	Persian Gulf
**Hap 3**	GQ355877	1	Persian Gulf
**Hap 4**	GQ355878	1	Persian Gulf
**Hap 5**	KF284059, KF284060, KF284061, KF284062, OR126983, OR126982, OR126981, OR676330, OR676331, OR676332, OR676333, OR676334, OR676335, OP056051, OP056046, OP056045, OP056039, OP056044, OP056049, OP056052, OP05604,2 OP056047, OP056048, OP056043, OP056040, OP056064, OP056065, OP056063, OP056061, OP056060, PX736885, PX736886, PX736887, PX736888, PX736890, PX736891, PX736892, PX736893, PX736894, PX736895, PX736896	41	Persian Gulf, Adriatic, East Med, Central Med and West Med
**Hap 6**	OR676328, OR676329	2	East and Central Med
**Hap 7**	OR676336	1	East Med
**Hap 8**	OR676337	1	East Med
**Hap 9**	PX736884, PX736889, PX736897, OR676338, OR676341, OR676342	6	Adriatic and Central Med
**Hap 10**	OP056050	1	West Med
**Hap 11**	OP056041	1	West Med
**Hap 12**	OP056062	1	West Med
**Hap 13**	OP056066	1	West Med

Table S5. The Adriatic *Pinctada radiata* ITS2 accession numbers from the present study, with their haplotypes as presented in the phylogenetic tree in Figure 4.

	**ITS2 Accession Numbers**
**Hap 1**	PX740674
**Hap 2**	PX740675
**Hap 3**	PX740676
**Hap 4**	PX740677, PX740688
**Hap 5**	PX740678, PX740679, PX740680, PX740682, PX740684, PX740687
**Hap 6**	PX740681
**Hap 7**	PX740683
**Hap 8**	PX740685
**Hap 9**	PX740686

References

Aguilo-Arce, J., Ferragut F., J., Png-Gonzalez, L., Carbonell, A., Capa, M., 2023. First genetic survey on the invasive rayed pearl oyster Pinctada radiata (Leach, 1814) populations of the Balearic Islands (Western Mediterranean). Mediterr. Mar. Sci. 24, 666–678. https://doi.org/10.12681/mms.34195

Arnaud, S., Monteforte, M., Galtier, N., Bonhomme, F., Blanc, F., 2000. Population structure and genetic variability of pearl oyster Pinctada mazatlanica along Pacific coasts from Mexico to Panama. Conserv. Genet. 1, 299–308. https://doi.org/10.1023/A:1011575722481

Barbieri, M., Deidun, A., Maltagliati, F., Castelli, A., 2016. A contribution to the phylogeography of Pinctada imbricata radiata (Leach, 1814) (Bivalvia: Pteriidae) from the Eastern Mediterranean Sea by means of the mitochondrial COI marker. Ital. J. Zool. 113–120. https://doi.org/DOI:%2010.1080/11250003.2015.1106012

Bouaziz-Yahiatene, H., Pfarrer, B., Medjdoub-Bensaad, F., Neubert, E., 2017. Revision of Massylaea Möllendorff, 1898 (Stylommatophora, Helicidae). ZooKeys 694, 109–133. https://doi.org/10.3897/zookeys.694.15001

Bratoš Cetinić, A., Bolotin, J., Grđan, S., 2023. Rayed pearl oyster Pinctada radiata (Leach, 1814) (Bivalvia: Pteriidae) in the eastern Adriatic Sea – recent observations. Naše More 70, 184–188. https://doi.org/10.17818/NM/2023/SI7

Christodoulou, M., Derycke, S., Beentjes, K.K., Hillewaert, H., Laakmann, S., Lundin, K., Kamyab, E., Khodami, S., Maes, S., Reiss, H., Uhlir, C., Van Den Bulcke, L., Van Der Hoorn, B., De Backer, A., Arbizu, P.M., 2025. A taxonomically reliable DNA barcode reference library for North Sea macrobenthos. Sci. Data 12, 1198. https://doi.org/10.1038/s41597-025-05500-z

Combosch, D.J., Collins, T.M., Glover, E.A., Graf, D.L., Harper, E.M., Healy, J.M., Kawauchi, G.Y., Lemer, S., McIntyre, E., Strong, E.E., Taylor, J.D., Zardus, J.D., Mikkelsen, P.M., Giribet, G., Bieler, R., 2017. A family-level Tree of Life for bivalves based on a Sanger-sequencing approach. Mol. Phylogenet. Evol. 107, 191–208. https://doi.org/10.1016/j.ympev.2016.11.003

Cunha, R.L., Blanc, F., Bonhomme, F., Arnaud-Haond, S., 2011. Evolutionary Patterns in Pearl Oysters of the Genus Pinctada (Bivalvia: Pteriidae). Mar. Biotechnol. 13, 181–192. https://doi.org/10.1007/s10126-010-9278-y

Doğan, A., Nerlović, V., 2008. On the occurrence of Pinctada radiata (Mollusca: Bivalvia: Pteriidae), an alien species in Croatian waters. Acta Adriat. 49, 155–158.

Feng, Y., Li, Q., Kong, L., 2015. Molecular phylogeny of Arcoidea with emphasis on Arcidae species (Bivalvia: Pteriomorphia) along the coast of China: Challenges to current classification of arcoids. Mol. Phylogenet. Evol. 85, 189–196. https://doi.org/10.1016/j.ympev.2015.02.006

Folmer, O., Black, M., Hoeh, W., Lutz, R., Vrijenhoek, R., 1994. DNA primers for amplification of mitochondrial cytochrome c oxidase subunit I from diverse metazoan invertebrates. Mol. Mar. Biol. Biotechnol. 3, 294–299.

Garzia, M., Doneddu, M., Giacobbe, S., Salvi, D., Trainito, E., Mariottini, P., 2024. Molecular and morphological data provide evidence for only one alien species of pearl oyster in the Mediterranean Sea. Sci. Mar. 88, e085. https://doi.org/10.3989/scimar.05432.085

Gavrilović, A., Piria, M., Guo, X.-Z., Jug-Dujaković, J., Ljubučić, A., Krkić, A., Iveša, N., Marshall, B.A., Gardner, J.P.A., 2017. First evidence of establishment of the rayed pearl oyster, Pinctada imbricata radiata (Leach, 1814), in the eastern Adriatic Sea. Mar. Pollut. Bull. 125, 556–560. https://doi.org/10.1016/j.marpolbul.2017.10.045

Gerovasileiou et al., C., 2017. New Mediterranean Biodiversity Records (July, 2017). Mediterr. Mar. Sci. 18, 355. https://doi.org/10.12681/mms.13771

He, M., Huang, L., 2023. Application of ITS-2 sequences of nuclear rDNA in phylogenetic studies of Pinctada (Mollusca: Bivalvia). J. Trop. Oceanogr. 22, 51–57.

He, M., Huang, L., Shi, J., Jiang, Y., 2005. Variability of Ribosomal DNA ITS-2 and Its Utility in Detecting Genetic Relatedness of Pearl Oyster. Mar. Biotechnol. 7, 40–45. https://doi.org/10.1007/s10126-004-0003-6

Institute Ruđer Bošković, 2025. First occurrence of the species Pinctada radiata (Mollusca: Bivalvia: Pteriidae) in a shellfish farm in the northern Adriatic Sea. URL https://www.irb.hr/Zavodi/Centar-za-istrazivanje-mora/Laboratorij-za-morsku-nanotehnologiju-i-biotehnologiju/Novosti/Prva-pojava-vrste-Pinctada-radiata-Mollusca-Bivalvia-Pteriidae-u-uzgajalistu-skoljkasa-u-sjevernom-Jadranu (accessed 10.23.25).

Katsanevakis, S., Zenetos, A., Mačić, V., Beqiraj, S., Poursanidis, D., Kashta, L., 2011. Invading the Adriatic: spatial patterns of marine alien species across the Ionian–Adriatic boundary. Aquat. Biol. 13, 107–118. https://doi.org/10.3354/ab00357

Lazoski, C., Gusmão, J., Boudry, P., Solé-Cava, A., 2011. Phylogeny and phylogeography of Atlantic oyster species: evolutionary history, limited genetic connectivity and isolation by distance. Mar. Ecol. Prog. Ser. 426, 197–212. https://doi.org/10.3354/meps09035

Lind, C.E., Evans, B.S., Elphinstone, M.S., Taylor, J.J.U., Jerry, D.R., 2012. Phylogeography of a pearl oyster (*Pinctada maxima*) across the Indo-Australian Archipelago: evidence of strong regional structure and population expansions but no phylogenetic breaks: Phylogeography of *Pinctada maxima*. Biol. J. Linn. Soc. 107, 632–646. https://doi.org/10.1111/j.1095-8312.2012.01960.x

Matsumoto, M., 2003. Phylogenetic analysis of the subclass Pteriomorphia (Bivalvia) from mtDNA COI sequences. Mol. Phylogenet. Evol. 27, 429–440. https://doi.org/10.1016/S1055-7903(03)00013-7

McIlroy, S.E., Guibert, I., Archana, A., Chung, W.Y.H., Duffy, J.E., Gotama, R., Hui, J., Knowlton, N., Leray, M., Meyer, C., Panagiotou, G., Paulay, G., Russell, B., Thompson, P.D., Baker, D.M., 2024. Life goes on: Spatial heterogeneity promotes biodiversity in an urbanized coastal marine ecosystem. Glob. Change Biol. 30, e17248. https://doi.org/10.1111/gcb.17248

Meyer, J.B., Cartier, L.E., Pinto-Figueroa, E.A., Krzemnicki, M.S., Hänni, H.A., McDonald, B.A., 2013. DNA Fingerprinting of Pearls to Determine Their Origins. PLoS ONE 8, e75606. https://doi.org/10.1371/journal.pone.0075606

Nerlović, V., Quinteiro, J., Rey-Méndez, M., 2017. A new record of the pearl oyster, Pinctada radiata (Bivalvia: Pteriidae), in the eastern Adriatic Sea (Croatia): morphological and molecular characterization, in: Foro Rec Mar Ac Rias Gal. Presented at the XXVIII Ciclo de Palestras: "Cultivando o Mar," Santos, Brazil, pp. 203–215.

Oh, C., Kim, J.-K., Son, Y.-B., Ju, S.-J., Jeung, H.-D., Yang, H.-S., Choi, K.-S., Le Moullac, G., Kang, D.-H., 2017. Phylogenetic, histological and age determination for investigation of non-native tropical black-lip pearl oyster, Pinctada margaritifera, settled in jeju, Korea. Ocean Sci. J. 52, 593–601. https://doi.org/10.1007/s12601-017-0054-z

Pagenkopp Lohan, K.M., Hill-Spanik, K.M., Torchin, M.E., Strong, E.E., Fleischer, R.C., Ruiz, G.M., 2015. Molecular phylogenetics reveals first record and invasion of Saccostrea species in the Caribbean. Mar. Biol. 162, 957–968. https://doi.org/10.1007/s00227-015-2637-5

Papadopoulos, D.K., Giantsis, I.A., Lattos, A., Triantafyllidis, A., Michaelidis, B., 2024. Marine bivalves voucher DNA barcoding from Eastern Mediterranean, with evidence for *Ostrea stentina* invasion. J. Mar. Biol. Assoc. U. K. 104, e46. https://doi.org/10.1017/S0025315424000377

Petović, M., Mačić, V., 2017. New data on Pinctada radiata (Leach, 1814) (Bivalvia: Pteriidae) in the Adriatic Sea. Acta Adriat. 58, 359–364.

Shan, B., Deng, Z., Ma, S., Sun, D., Liu, Y., Yang, C., Wu, Q., Yu, G., 2023. A New Record of Pinctada fucata (Bivalvia: Pterioida: Pteriidae) in Mischief Reef: A Potential Invasive Species in the Nansha Islands, China. Diversity 15, 578. https://doi.org/10.3390/d15040578

Sharif Ranjbar, M., Zolgharnien, H., Yavari, V., Archangi, B., Ali Salari, M., Arnaud-Haond, S., Cunha, R.L., 2016. Rising the Persian Gulf Black-Lip Pearl Oyster to the Species Level: Fragmented Habitat and Chaotic Genetic Patchiness in Pinctada persica. Evol. Biol. 43, 131–143. https://doi.org/10.1007/s11692-015-9356-1

Vio, E., De Min, R., 1996. Contributo alla conoscenza dei Molluschi marini del Golfo di Trieste. Atti del Museo Civico di Storia Naturale di Trieste 47.

Yu, D.H., Chu, K.H., 2006. Species identity and phylogenetic relationship of the pearl oysters in Pinctada Röding, 1798 based on ITS sequence analysis. Biochem. Syst. Ecol. 34, 240–250. https://doi.org/10.1016/j.bse.2005.09.004

Yu, D.H., Jia, X., Chu, K.H., 2006. Common pearl oysters in China, Japan, and Australia are conspecific: evidence from ITS sequences and AFLP. Fish. Sci. 72, 1183–1190. https://doi.org/10.1111/j.1444-2906.2006.01275.x
